# Supplementary material for: Trans-eQTL hotspots shape complex traits by modulating cellular states
Source: Cell Genom. 2025 May 5;5(5):100873. doi: 10.1016/j.xgen.2025.100873 (PMC12143327; doi:10.1016/j.xgen.2025.100873)
Supplement: Document S1. Figures S1–S16 [file mmc1.pdf]

Cell Genomics, Volume *5*

Supplemental information

Trans-eQTL hotspots shape complex traits by modulating cellular states

Kaushik Renganaath and Frank Wolfgang Albert

Supplementary figures

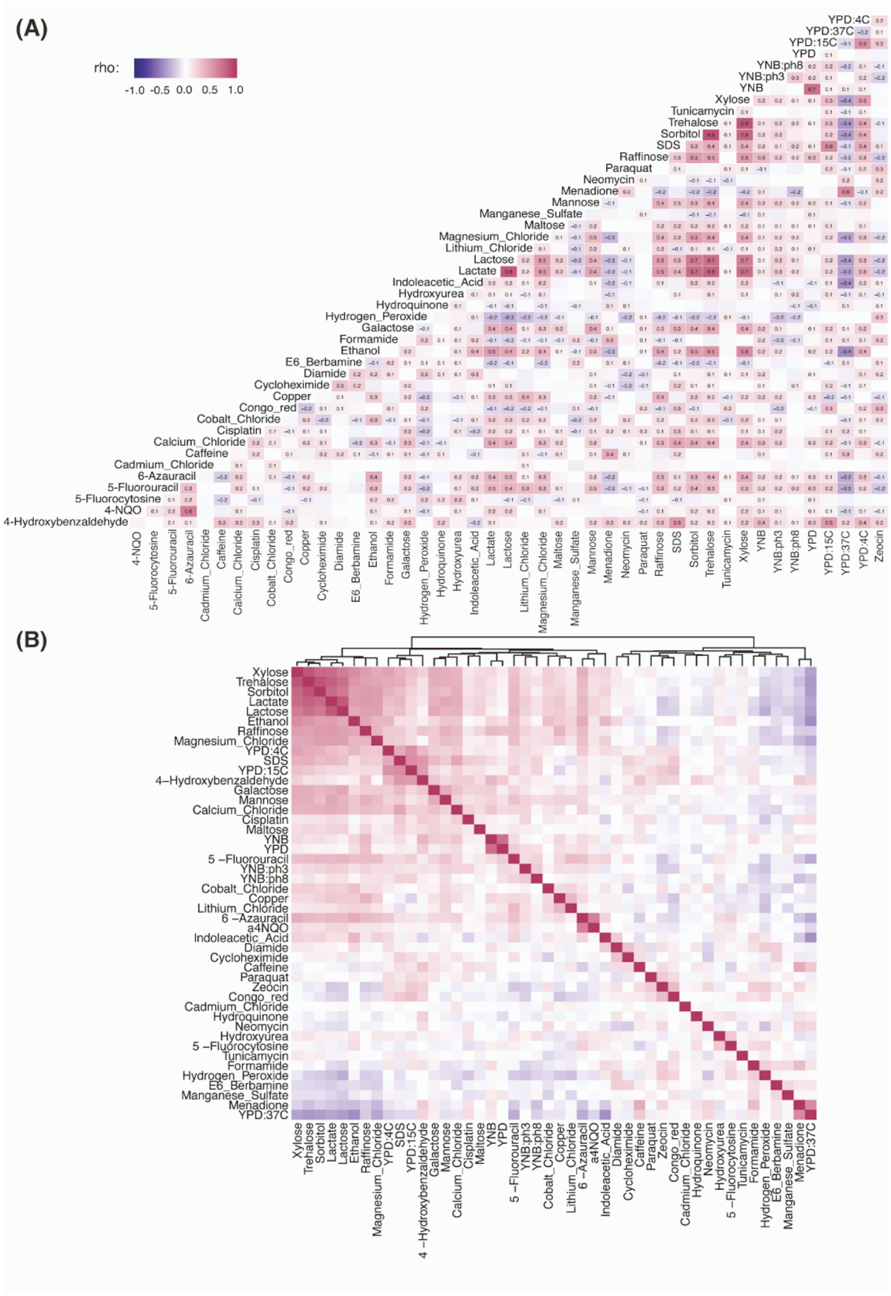

Figure S1. **Growth trait correlations**, *related to Figure 1*. (A) Spearman correlation coefficients (rho) for all pairs of traits. Significant spearman correlation coefficients are displayed within the cells of the heatmap. (B) Clustering of traits based on the correlation of growth of 979 segregants in the respective conditions.

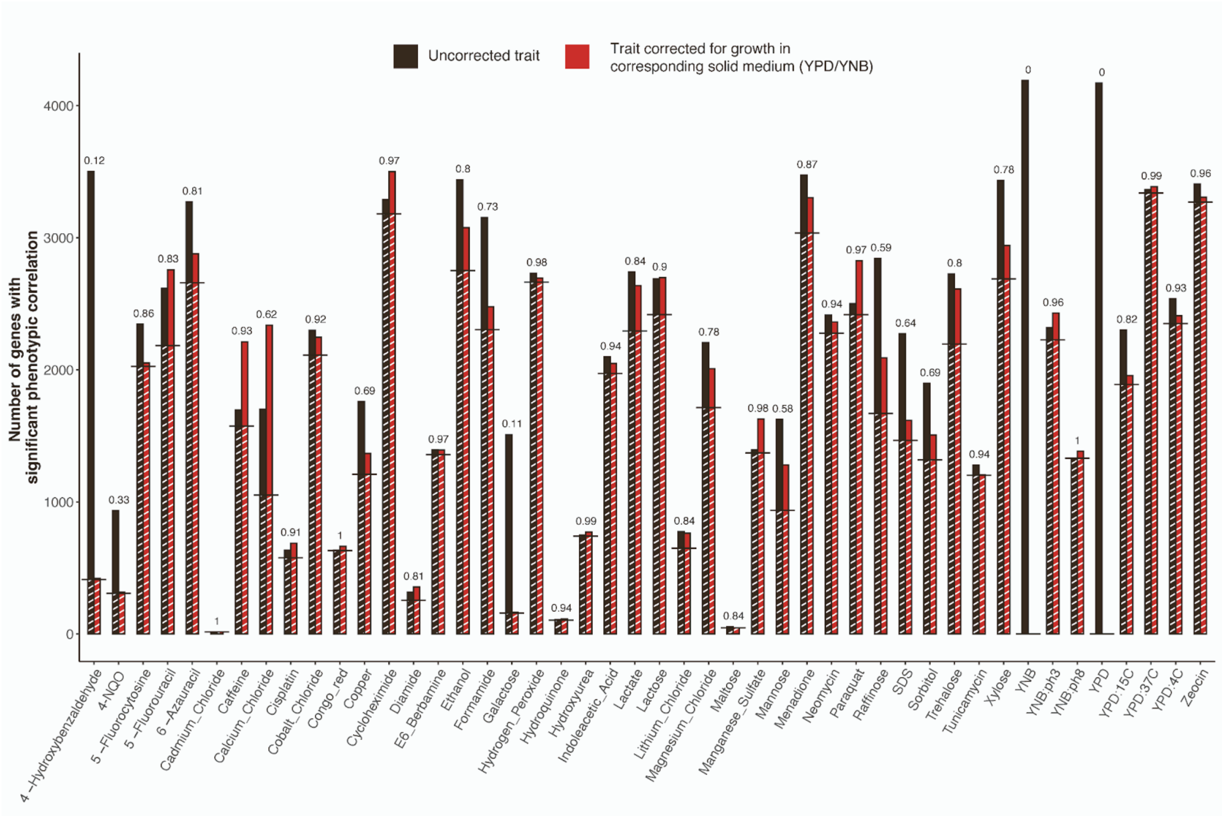

Figure S2. **Number of genes with significant genetic correlations at 5% FDR before (black) and after (red) correcting for growth on the respective solid agar medium (YPD or YNB)**, *related to Figure 1*. White shading and black horizontal lines show genes that have significant correlations before as well as after correction. Numbers above each pair of black and red bars indicate the fraction of genes with a genetic correlation that persisted after correction for the solid medium. Correcting for growth on the solid agar medium eliminated all correlations for YNB and YPD. For three traits, more than half of the correlations lost significance: galactose: 90%, 4-Hydroxybenzaldehyde: 89%, 4-Nitroquinoline oxide: 68%. For the remaining 41 traits, a median of 11% of correlations became non-significant.

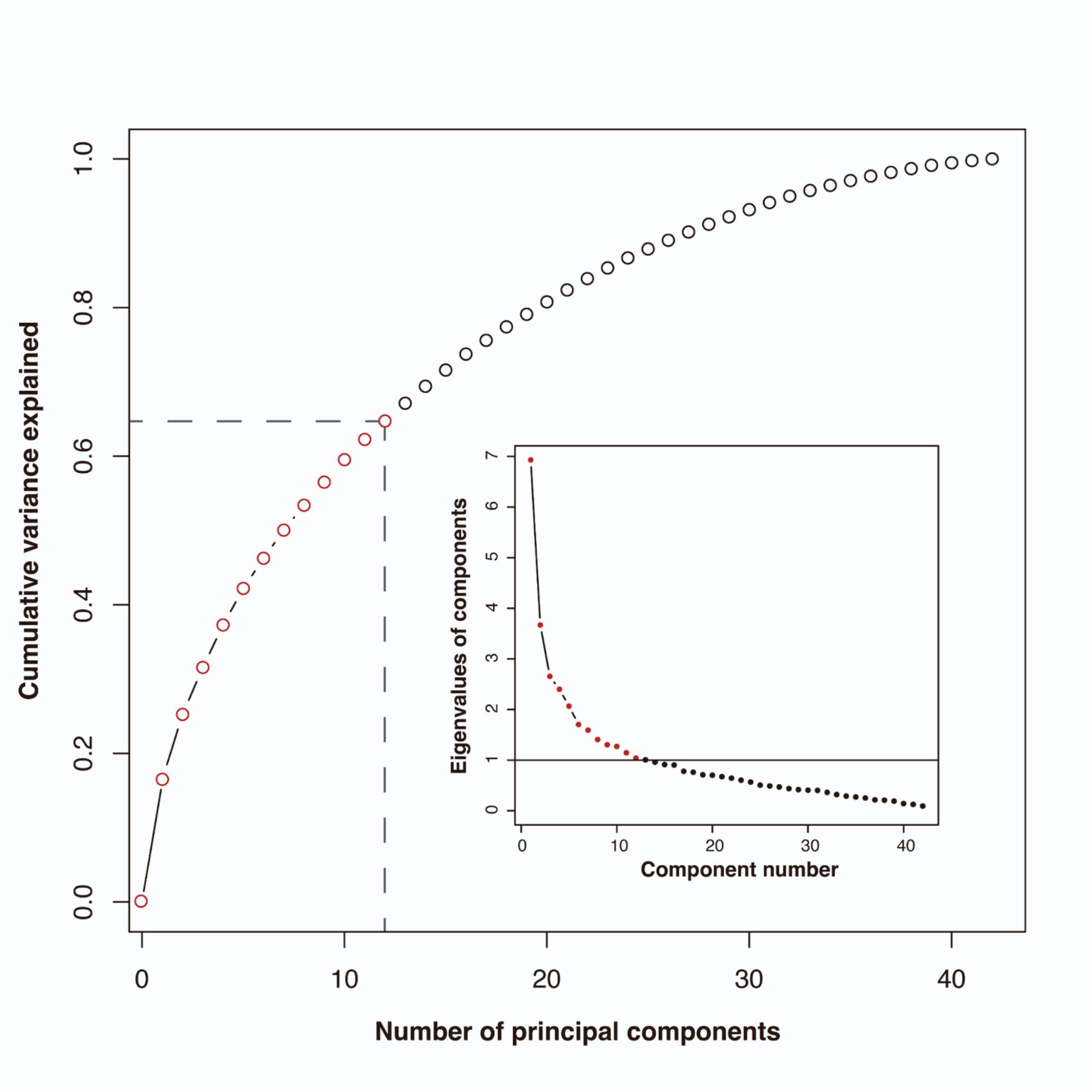

Figure S3. **Cumulative distribution of the proportion of variance among 42 growth traits explained by principal components**, *related to Figure 1*. These analyses excluded 4 traits with high missing data (Table S1). The inset shows a scree plot for the same principal component analysis. The first 12 principal components account for most of the variance in growth traits based on the Kaiser criterion (Eigenvalue of component ≥ 1, points indicated in red) [1]. Together, these 12 components account for ~65% of the variance in growth traits.

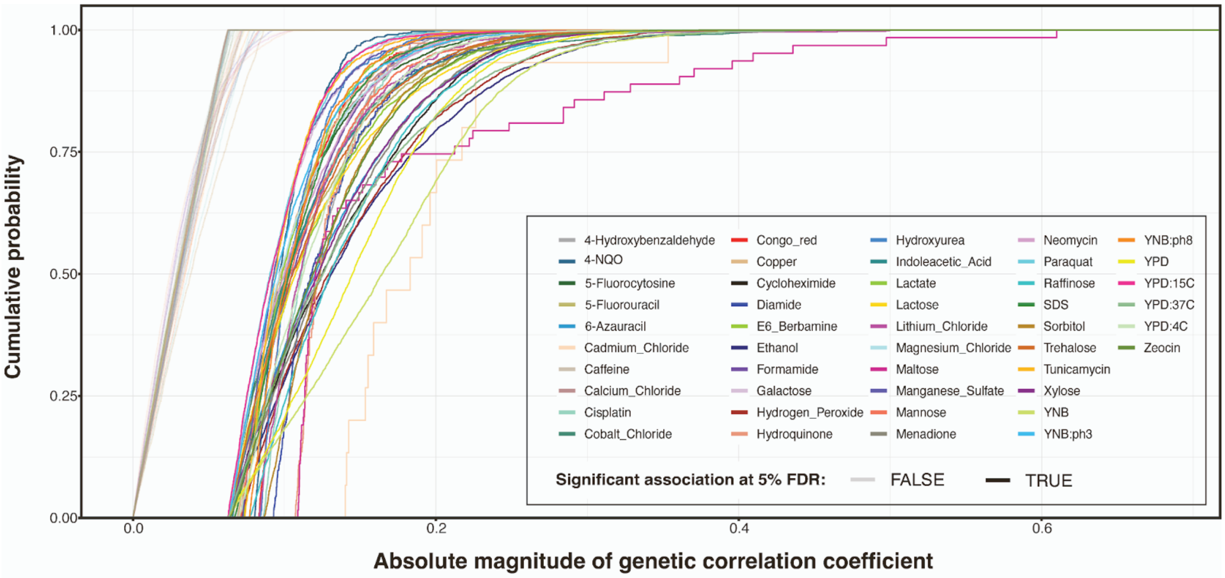

Figure S4. **Cumulative distribution of absolute magnitudes of correlation coefficients for genetic correlations between gene expression and growth in each of the 46 conditions**, *related to Figure 1*. For each condition, the figure shows separate distributions for significant (FDR of 5%) correlations (curves in saturated colors) and non-significant correlations (curves in pale colors). Most of the genetic correlations between gene expression and traits had modest magnitudes, with an overall median absolute correlation coefficient of 0.11.

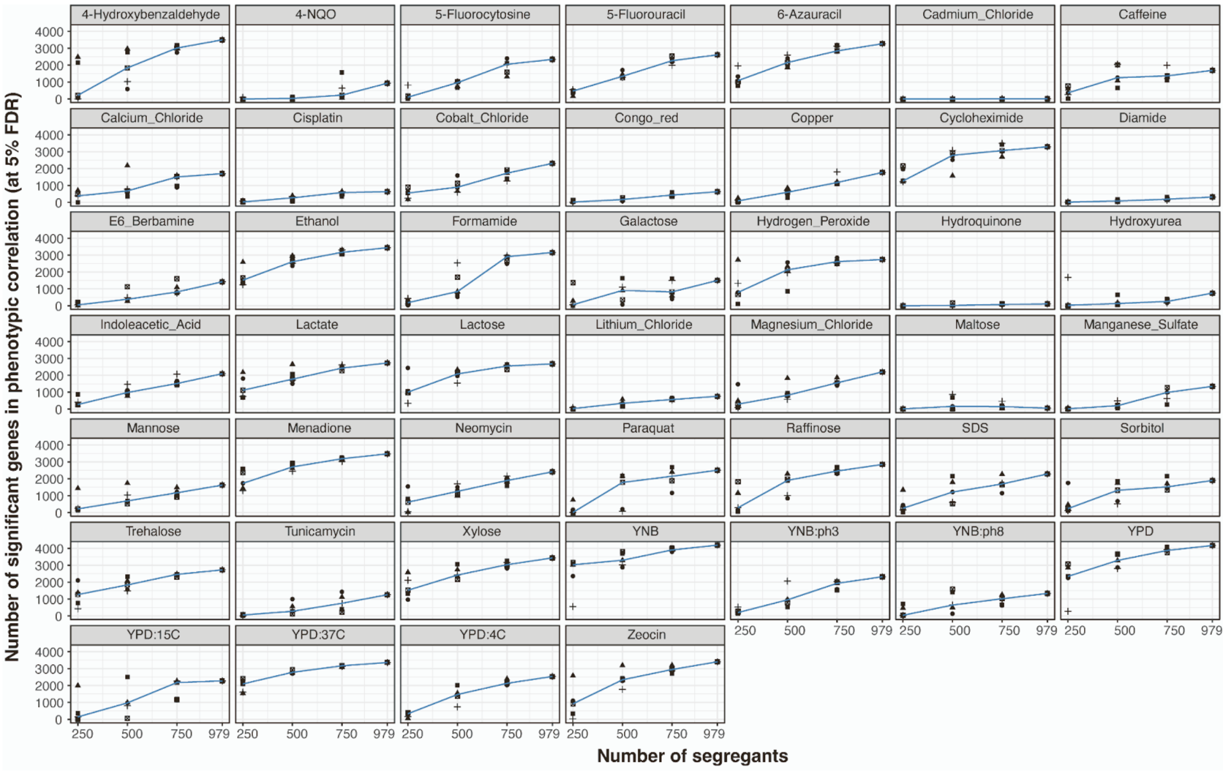

Figure S5. **The number of genes with significant correlation (5% FDR) between expression and growth in different conditions as a function of sample size**, *related to Figure 1*. We performed five random draws per sample size, indicated by different symbols. The trend line represents the median number of genes with significant correlation across the five draws for a given sample size.

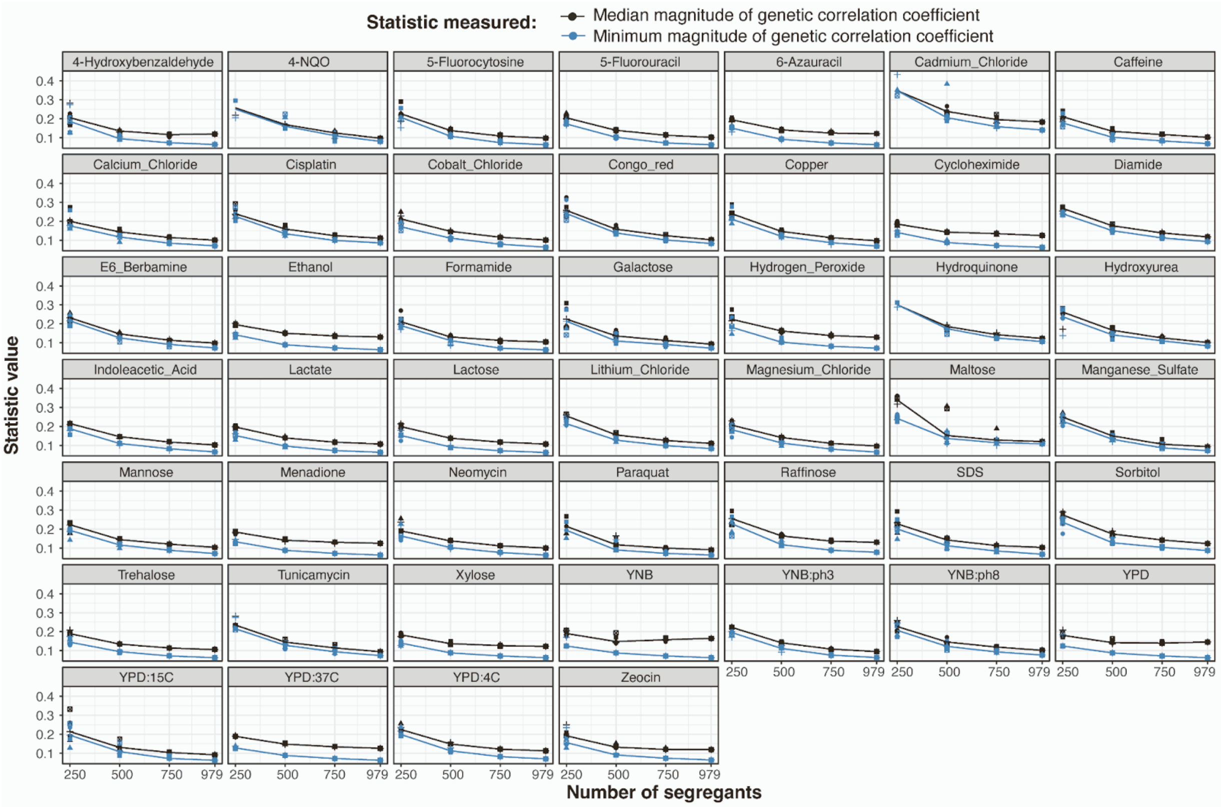

Figure S6. **Medians and minimums of the magnitudes of the correlation coefficients for significant correlations between gene expression and growth as a function of sample size,** *related to Figure 1*. We performed five random draws per sample size, indicated by different symbols. The trend line represents the median value of the statistic across the five draws for a given sample size. The median magnitude of the detected significant associations decreased with increasing sample size, as expected for larger samples that can detect correlations of weaker magnitude (see also Figure S5).

3

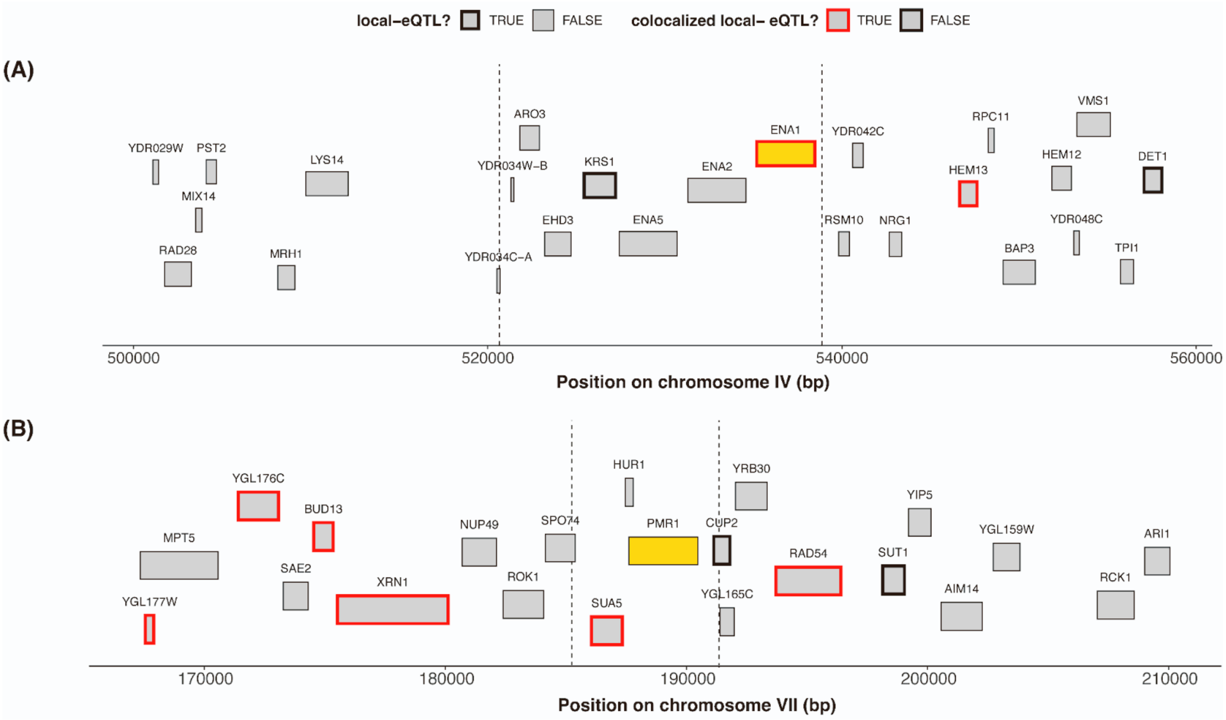

Figure S7. **Examples of local eQTLs at gQTLs with likely (top) and demonstrated (bottom) causal genes,** *related to Figure 2*. Show are chromosome regions with genes as boxes. Causal genes are shown in yellow. Dotted lines show 95% confidence intervals of gQTL location. Genes with a local eQTL with LOD ≥ 10 and a confidence interval that overlaps the gQTL have bold outlines. Genes without a local eQTL have thin outlines. Genes whose local eQTL is colocalized with the gQTL have red outlines. Genes whose local eQTL is not colocalized with the gQTL (test for two separate QTLs, $p < 0.05$) have black outlines. (A) A gQTL for growth in the presence of lithium chloride. *ENA1* is the likely causal gene for this gQTL, as variable copy number at the *ENA* locus underlies yeast growth variation [2,3]. Three local eQTLs overlapped with this gQTL, two of which (at *ENA1* and *HEM13*) were classified as colocalized. The *ENA1* local eQTL is extremely strong (LOD = 359, r = -0.89), as expected if higher *ENA1* expression from the BY allele is caused by a higher number of expressed copies in this strain (note that ENA copy number numbers in the strains analyzed here are not known). Indeed, *ENA1* is flagged as having a colocalized eQTL, suggesting that colocalization analyses can detect cases of causal colocalization, especially when the underlying effects are strong. *HEM13* is a likely false positive. (B) A gQTL for growth in the presence of manganese sulfate. A missense variant in *PMR1* has been experimentally demonstrated to cause this gQTL [4]. *PMR1* does not have a local eQTL and therefore cannot be detected by this colocalization analysis. Instead, local eQTLs at six other genes were flagged as colocalized with this gQTL (red boxes). While we cannot rule out that the causal variants creating these six local eQTLs contribute minor effects to this gQTL, it seems likely that these eQTLs were incorrectly flagged as colocalized due to linkage with the causal missense variant in *PMR1*.

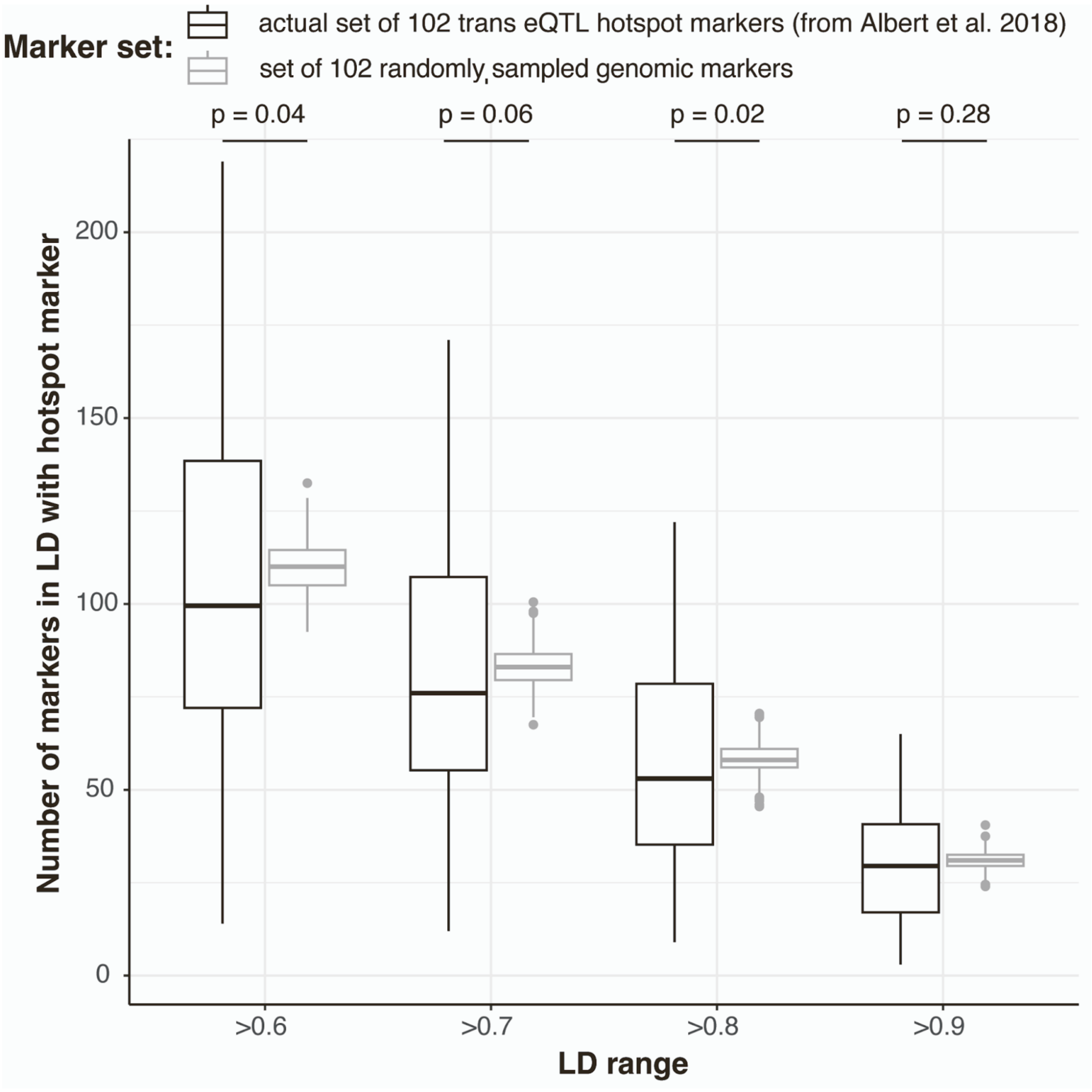

Figure S8. **Boxplots comparing the number of markers in LD with the 102 trans eQTL hotspot markers and 102 random markers at different LD ranges**, *related to Figure 5*. The values for the light gray boxes are the median of the number of markers for each of the 1000 random sets of 102 markers in the genome at the indicated LD ranges. LD between two markers is computed as the Pearson correlation coefficient between the genotypes at both markers.

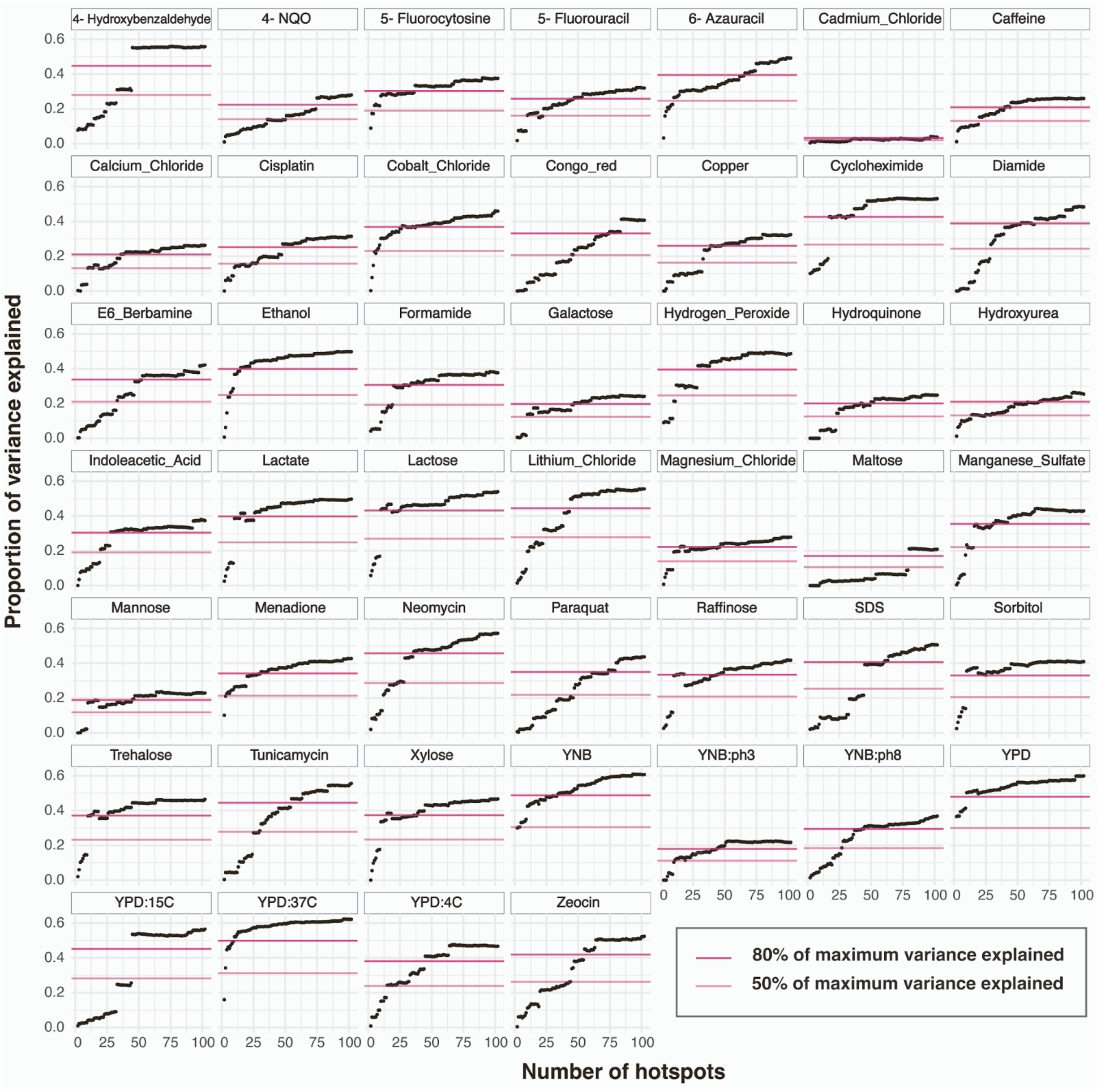

Figure S9. **Proportion of growth variance explained by top trans-eQTL hotspots**, related to *Figure 5.* The trans eQTL hotspots are ranked from 2 to 102 according to the number of genes whose expression they affect, for each of the 46 growth conditions. The values corresponding to 80% and 50% of the maximum variance explained by the trans eQTL hotspot sets for each of the growth conditions are indicated by the dark pink and light pink lines, respectively.

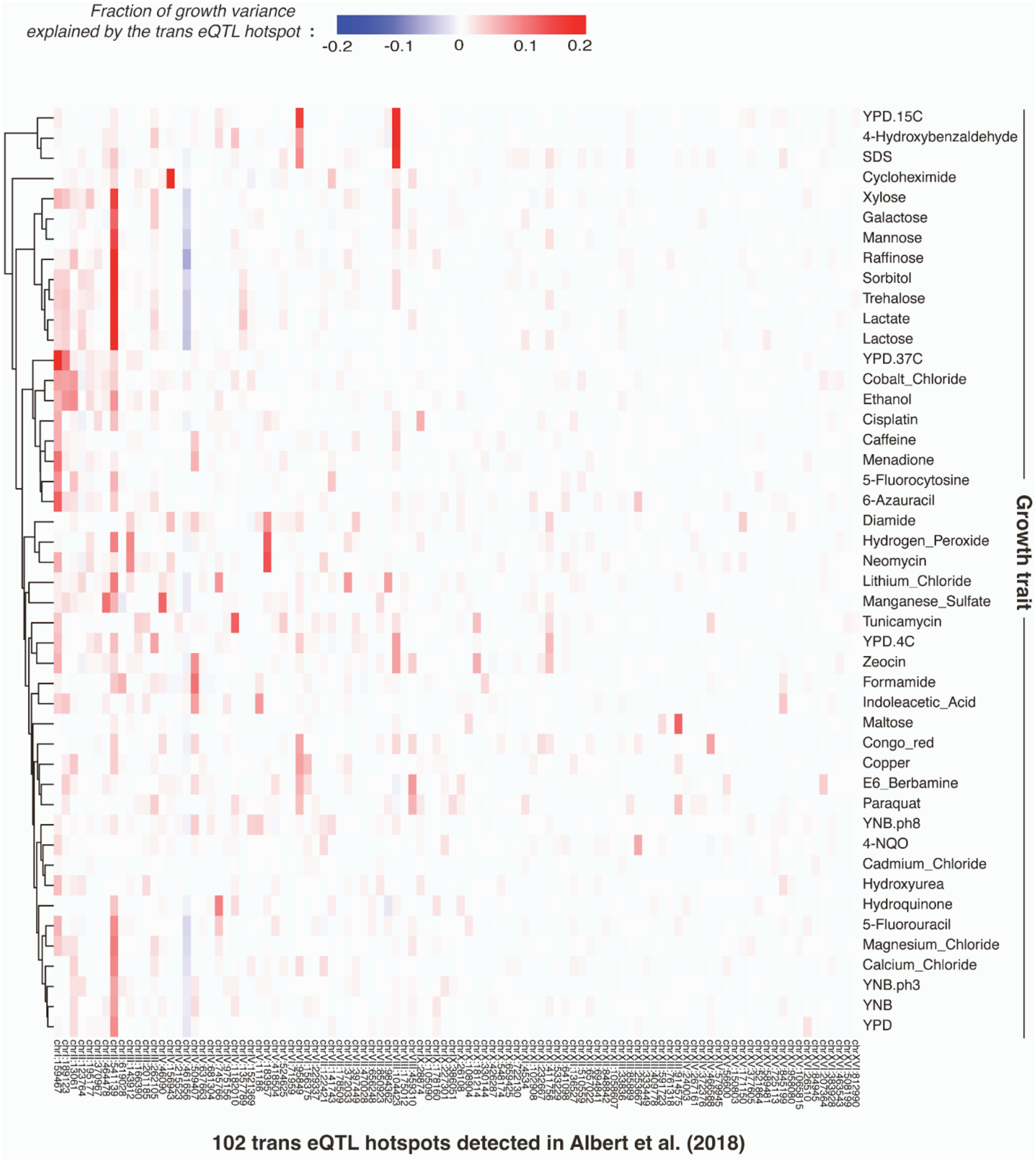

Figure S10. **Proportion of phenotypic variance explained by each of the 102 trans eQTL hotspots detected by Albert et al [5] for the 46 growth traits,** *related to Figure 5*.

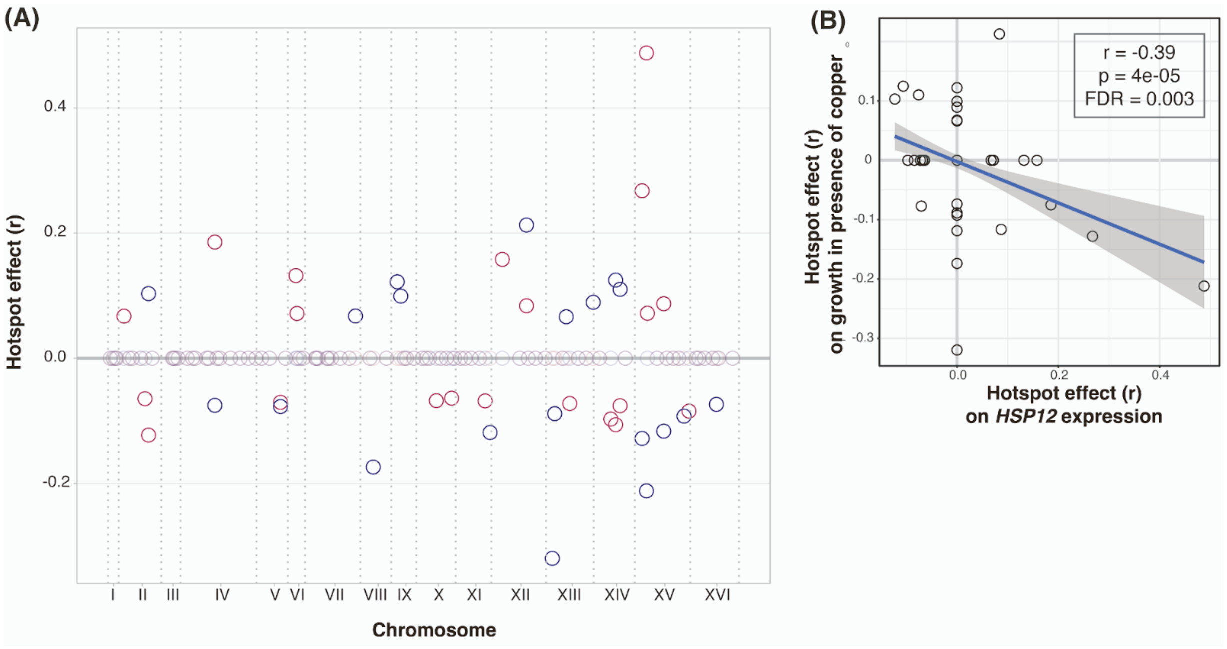

Figure S11. **Correlation of *trans*-eQTL hotspot effects on expression of *HSP12* and on growth in the presence of copper,** *related to Figure 5*. (A) The plot shows the genomic locations of the *trans*-eQTL hotspots and their effect on the expression of *HSP12* (red circles) and on growth in the presence of copper (blue circles). Effects are coefficients of correlation between trait and genotype at the hotspot marker. Pale circles at the zero line indicate hotspot effects estimated as zero (Methods). (B) The scatterplot shows the effects of *trans*-eQTL hotspots on *HSP12* expression and on growth in the presence of copper, as shown in A. The regression line along with its 95% confidence interval is also displayed.

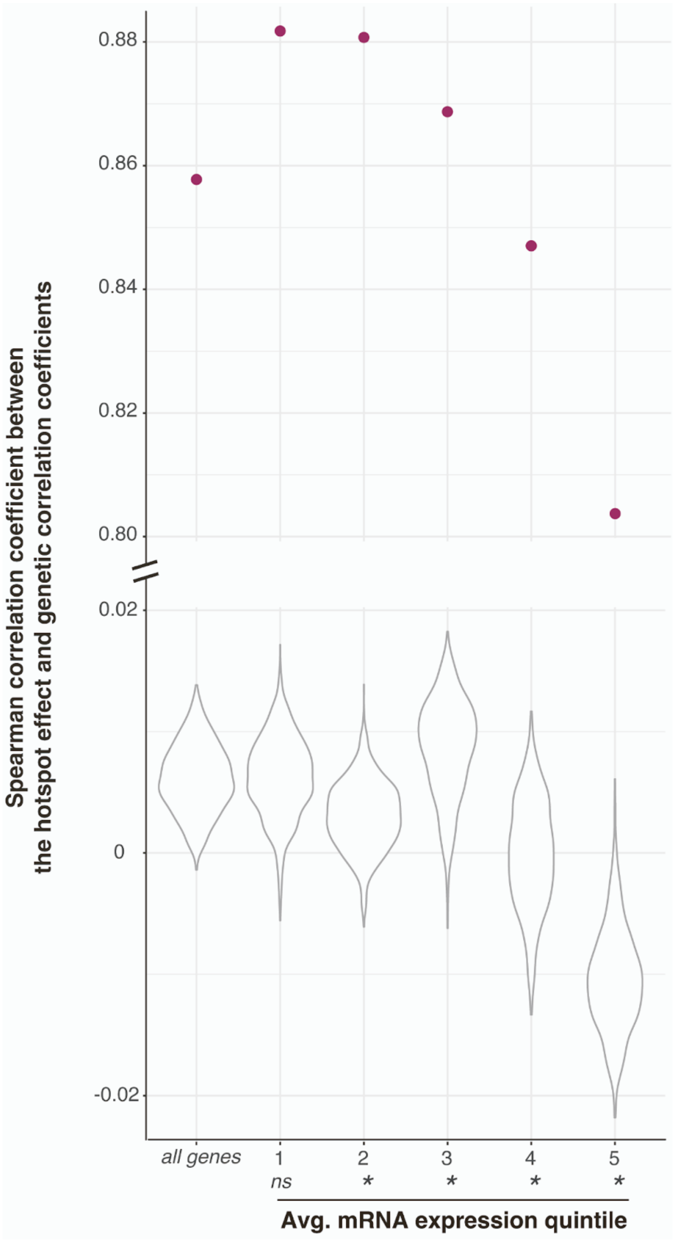

Figure S12. **Spearman correlation coefficients between hotspot effects and genetic correlation coefficients across all conditions and genes, stratified by expression quintiles,** *Figure 5*. Violin plots illustrate the distribution of correlation coefficients for 1,000 randomly generated sets of 102 markers. The actual correlation coefficients for the 102 hotspot markers are represented as pink dots. (Note: The result for "all genes" corresponds to Figure 5B.) The X-axis also indicates whether the mean correlation coefficients for the random sets within each quintile bin are significantly different from the "all genes" set, as determined by Wilcoxon tests ($p < 0.05$): ns = not significant, * = significant.

13

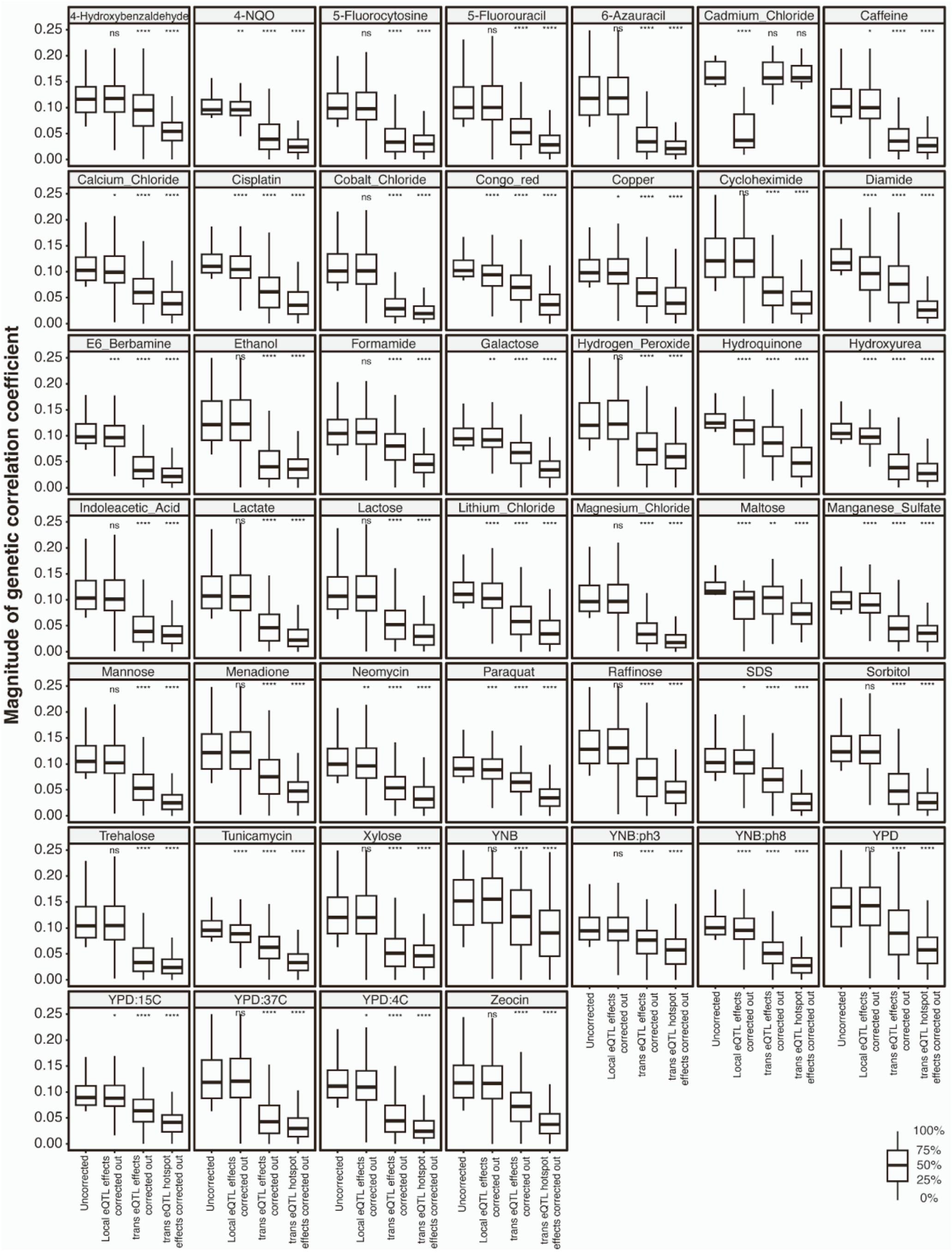

Figure S13. **Distributions of magnitudes of genetic correlation coefficients before and after correcting for the effects of local eQTLs, *trans*-eQTLs and *trans*-eQTL hotspots,** *related to Figure 5*. Descriptions of the different categories are as in Figure 5C. The significance of the difference between the medians of the distributions with respect to the 'uncorrected' category was computed using the Wilcoxon test and significance indicated as follows: *ns* - not significant; * 0.01 < p < 0.05; ** p < 0.01; *** p < 0.001; **** p < 1e-04.

14

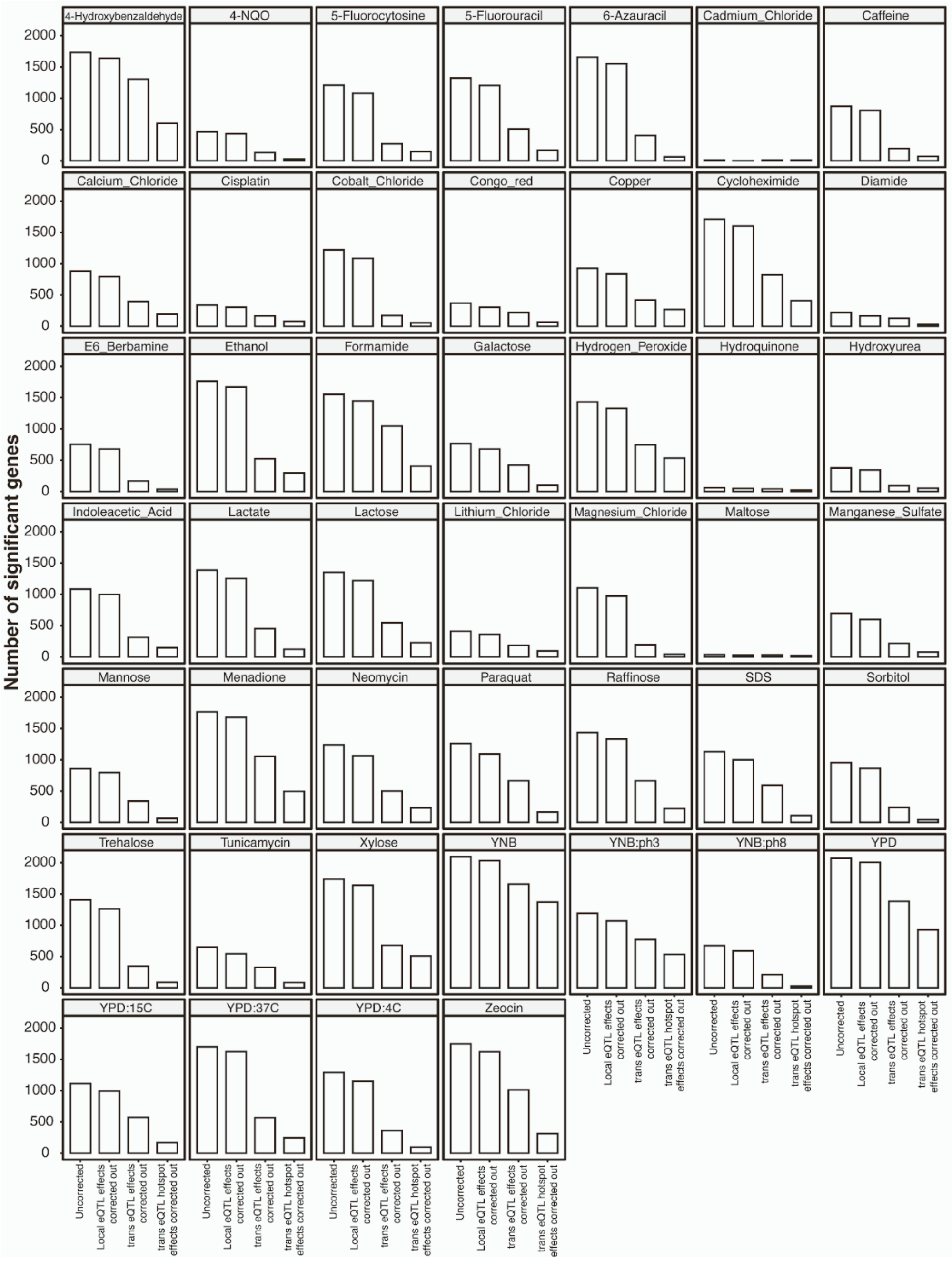

Figure S14. **Number of genes with significant genetic correlation before and after correcting for the effects of local eQTLs, *trans*-eQTLs and *trans*-eQTL hotspots,** *related to Figure 5*. The ‘uncorrected’ category contains genes with significant genetic correlation at 5% FDR with at least one local eQTL and at least one *trans*-eQTL. Out of these genes, the number of genes with significant genetic correlation at nominal $p < 0.05$ after correcting out the effects of these genes’ local eQTLs, their *trans*-eQTLs, and the 102 *trans*-eQTL hotspots is represented.

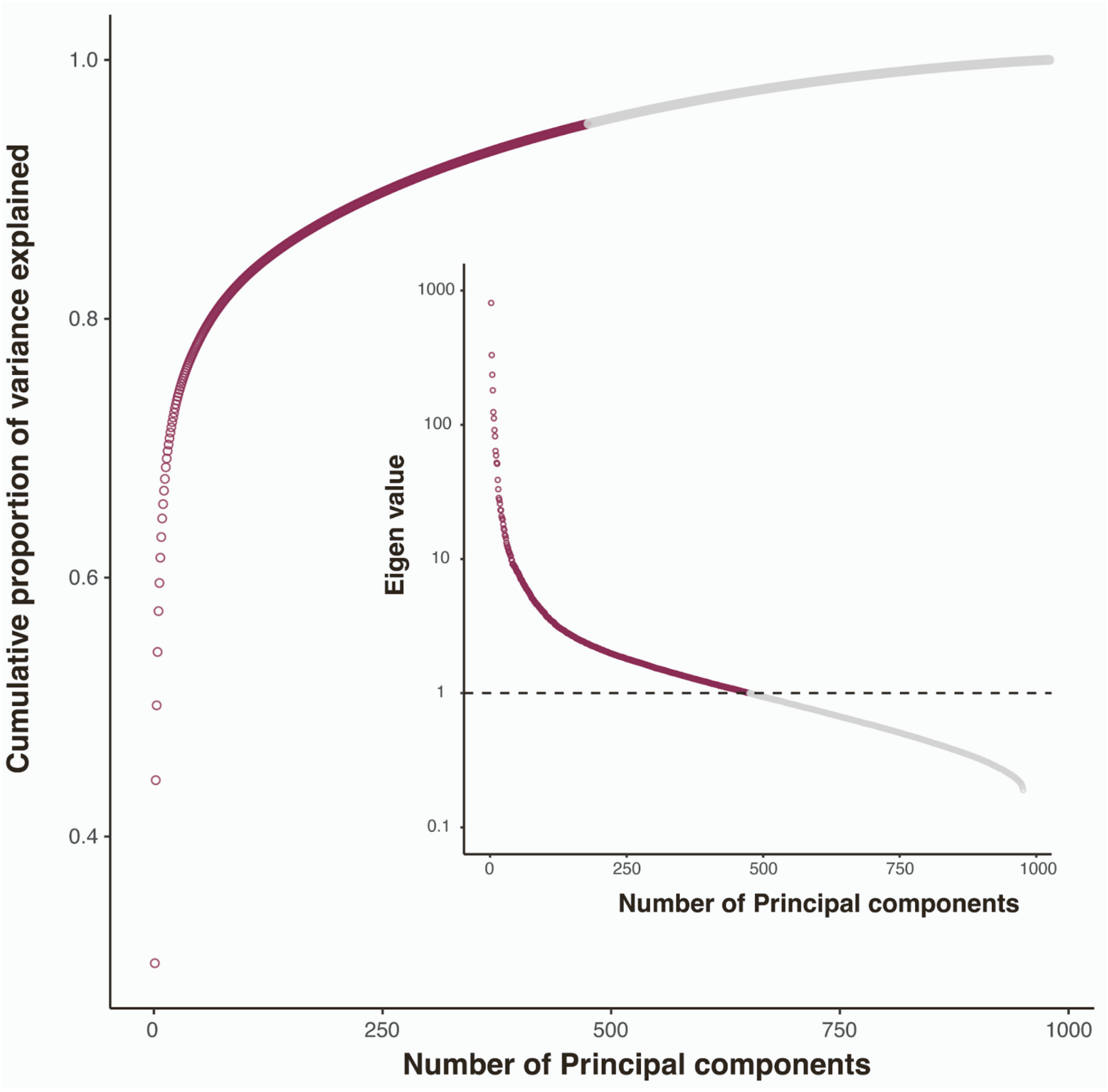

Figure S15. **Cumulative distribution of the proportion of variance among gene expression (for 5720 genes assayed by Albert et al. [5]) explained by principal components,** *related to STAR Methods*. The inset shows a scree plot for the same principal component analysis. For principal components with Eigenvalue ≥ 1, points are indicated in dark pink.

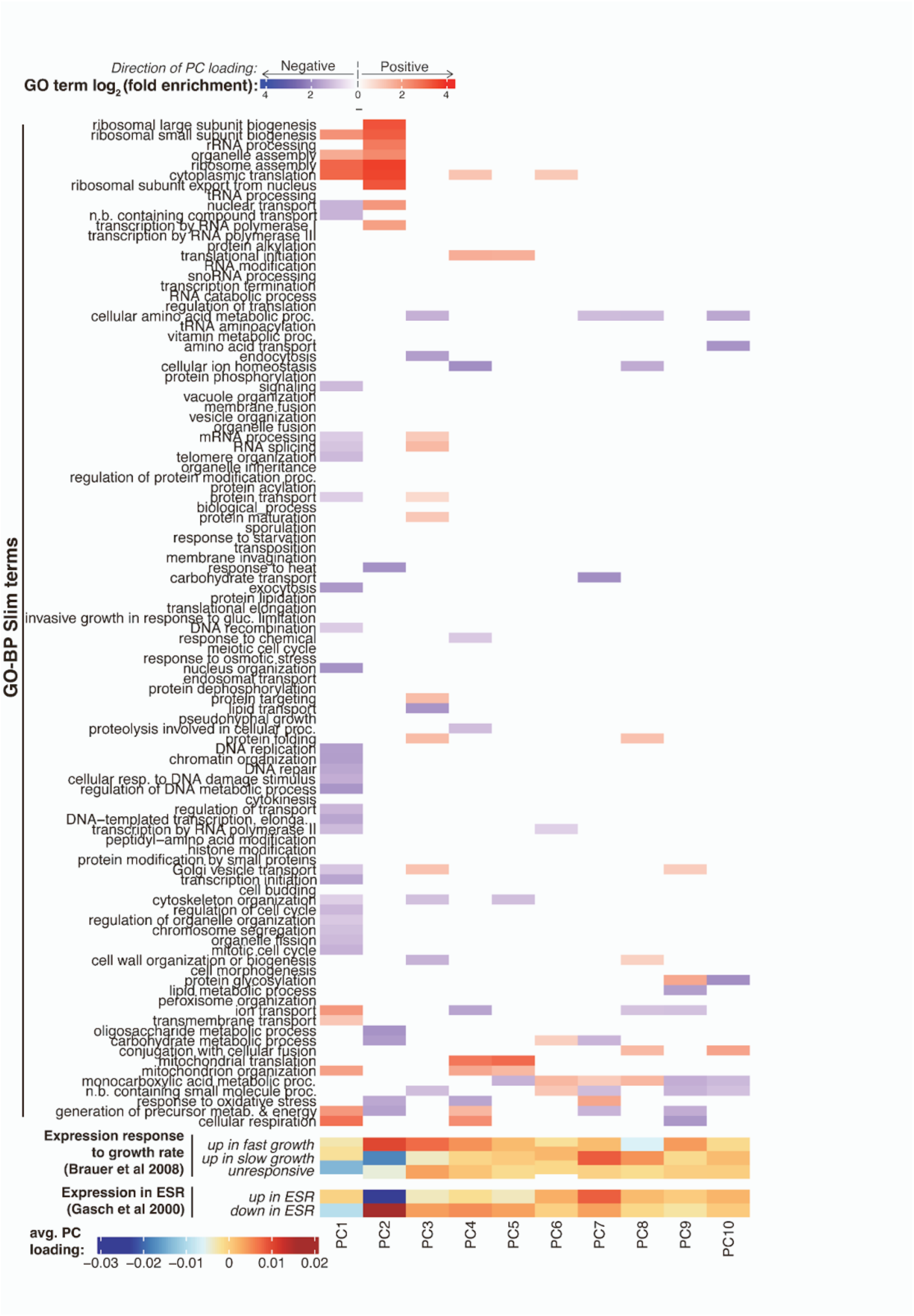

Figure S16. **Biological processes enriched in expression PCs,** *related to STAR Methods*. The top heatmap shows significant log2-fold enrichments (uncorrected p < 0.001) for 100 GO-BP Slim terms in the top 10% set of genes that load most strongly in both directions (negative indicated by blue and positive indicated in red). The heatmaps at the bottom show average PC loadings for genes in the indicated gene groups from Brauer et al. [6] and Gasch et al [7].

Supplementary references

1. Kaiser, H.F. (1960). The Application of Electronic Computers to Factor Analysis. Educ. Psychol. Meas. *20*, 141–151. https://doi.org/10.1177/001316446002000116.

2. Treusch, S., Albert, F.W., Bloom, J.S., Kotenko, I.E., and Kruglyak, L. (2015). Genetic Mapping of MAPK-Mediated Complex Traits Across S. cerevisiae. PLOS Genet. *11*, e1004913. https://doi.org/10.1371/journal.pgen.1004913.

3. Warringer, J., Zörgö, E., Cubillos, F.A., Zia, A., Gjuvsland, A., Simpson, J.T., Forsmark, A., Durbin, R., Omholt, S.W., Louis, E.J., et al. (2011). Trait Variation in Yeast Is Defined by Population History. PLOS Genet. *7*, e1002111. https://doi.org/10.1371/journal.pgen.1002111.

4. Sadhu, M.J., Bloom, J.S., Day, L., and Kruglyak, L. (2016). CRISPR-directed mitotic recombination enables genetic mapping without crosses. Science *352*, 1113–1116. https://doi.org/10.1126/science.aaf5124.

5. Albert, F.W., Bloom, J.S., Siegel, J., Day, L., and Kruglyak, L. (2018). Genetics of trans-regulatory variation in gene expression. eLife *7*, e35471. https://doi.org/10.7554/eLife.35471.

6. Brauer, M.J., Huttenhower, C., Airoldi, E.M., Rosenstein, R., Matese, J.C., Gresham, D., Boer, V.M., Troyanskaya, O.G., and Botstein, D. (2008). Coordination of Growth Rate, Cell Cycle, Stress Response, and Metabolic Activity in Yeast. Mol. Biol. Cell *19*, 352–367. https://doi.org/10.1091/mbc.e07-08-0779.

7. Gasch, A.P., Spellman, P.T., Kao, C.M., Carmel-Harel, O., Eisen, M.B., Storz, G., Botstein, D., and Brown, P.O. (2000). Genomic Expression Programs in the Response of Yeast Cells to Environmental Changes. Mol. Biol. Cell *11*, 4241–4257. https://doi.org/10.1091/mbc.11.12.4241.
